# CNP Signal Peptide in Patients with Cardiovascular Disease

**DOI:** 10.3389/fcvm.2015.00028

**Published:** 2015-06-15

**Authors:** Jacqui Lee, Martin Than, Sally Aldous, Richard Troughton, Mark Richards, Chris J. Pemberton

**Affiliations:** ^1^Department of Medicine, Christchurch Heart Institute, University of Otago, Christchurch, New Zealand

**Keywords:** C-type natriuretic peptide, chest pain, signal peptide, myocardial infarction, atrial fibrillation

## Abstract

We have previously reported that signal peptide fragments of C-type natriuretic peptide (CNP) are present in the human circulation. Here, we provide the first preliminary assessment of the potential utility of CNP signal peptide (CNPsp) measurement in acute cardiovascular disease. Utilizing our specific and sensitive immunoassay, we assessed the potential of CNPsp measurement to assist in the identification of acute coronary syndromes in 494 patients presenting consecutively with chest pain. The diagnostic and prognostic potential of CNPsp were assessed in conjunction with a contemporary clinical troponin I assay, an investigational highly sensitive troponin T assay and NT-proBNP measurement. Utility was assessed via receiver operator curve characteristic analysis. CNPsp did not identify patients with myocardial infarction (MI) or those with unstable angina, nor did it assist the diagnostic ability of clinical or investigational troponin measurement. CNPsp levels were significantly elevated in patients presenting with atrial fibrillation (*P* < 0.05) and were significantly lower in those with a history of previous MI (*P* < 0.05). CNPsp could identify those at risk of mortality within 1 year (*P* < 0.05) and also could identify those at risk of death or re-infarction within 1 year (*P* < 0.01). This is the first exploratory report describing the potential of CNPsp measurement in acute cardiovascular disease. While CNPsp does not have utility in acute diagnosis, it may have potential in assisting risk prognosis with respect to mortality and re-infarction.

## Introduction

We have recently reported that signal peptide (SP) fragments derived from each of A-type natriuretic peptide (ANP), B-type natriuretic peptide (BNP), and C-type natriuretic peptide (CNP) are present in the human circulation (1–3). Each of these fragments derives from the carboxyl terminus of their respective signal peptide and all of them bear biochemical adducts of differing sizes. In normal healthy individuals, plasma levels of each SP do not correlate with their propeptide siblings and they also respond differently during the course of ST-elevation myocardial infarction (STEMI). All three SP do show evidence of cardiac secretion and this raises the possibility that their measurement in plasma may have utility in cardiovascular disease.

Whereas circulating concentrations of ANP and BNP are predominately influenced by cardiac secretion (1, 2), CNP gene-derived peptides appear to be secreted from multiple organs and are thought to be more reliant on general vascular release (4). By contrast, our previous report (3) suggested that plasma concentrations of CNP signal peptide (CNPsp) may be more reliant on cardiac and renal production and that plasma levels of CNPsp were raised in patients suffering STEMI. Given this background, we now report preliminary, exploratory findings on CNPsp measurement in patients with chest pain suggestive of an acute coronary syndrome (ACS), presenting to single center, hospital emergency department. The aim of this work was to determine if CNPsp measurement might have potential utility in the diagnosis and/or prognosis of patients with chest pain.

## Materials and Methods

### Blood sample collection

#### Prospective Chest Pain Study

For this study, patients presenting to Christchurch Hospital with the primary complaint of chest pain <4 h duration were offered recruitment into our prospective, observational study known as signal peptides in acute coronary events (SPACE, http://www.anzctr.org.au, ACTRN12609000057280). Patients with the primary complaint of acute chest, epigastric, neck, jaw, or arm pain suspicious of ACS, without obvious non-cardiac origin, and lasting ≥20 min were enrolled in accord with guideline definitions (5). More general/atypical symptoms (such as fatigue, nausea, vomiting, sweating, and faintness) were not used as inclusion criteria and those on dialysis or with terminal kidney failure were excluded. EDTA blood samples for measurement of hsTnT, proBNP, and CNPsp and Heparin blood samples for measurement of TnI were taken at time 0, 1, 2, and 12–24 h after presentation. The adjudicated diagnosis of acute MI was made in accordance with the 2012 ESC/ACCF/AHA/WHF taskforce guidelines (6), by two independent cardiologists with access to all clinical data but not CNPsp or hsTnT. The biochemical component of the diagnosis of MI was made using a late generation TnI assay with 1 value ≥99th URL (99th percentile = 0.03 μg/L) and a rise or fall of 50% of the URL (=0.015 μg/L) within 12 h of presentation.

### Follow up and prognostic end points

At 45 and 365 days post-discharge from hospital, consented and enrolled patients in the ACS study were contacted by telephone or in writing to complete a follow up interview/questionnaire. Reported clinical events were identified from the patients themselves (or their primary physician) and confirmed by clinical adjudication, centralized New Zealand Ministry of Health database registry entries on mortality and events and with records of the treating institution. The prognostic end points considered were mortality, subsequent MI, subsequent episode of acute decompensated heart failure (ADHF), and subsequent stroke. We also considered the composite end point of mortality or MI within 365 days.

### CNPsp and cardiac marker assays

CNP signal peptide was measured using our in-house immunoassay, as previously reported (3). Briefly, extracted plasma samples and standards were diluted in assay buffer, with the assay incubate consisting of 50 μL of sample or standard (0–3,630 pmol/L of CNPsp peptide) mixed with 50 μl of antibody at 1:8000 dilution and left to incubate for 22 h at 4°C. Fifty microliters of iodinated CNPsp(Tyr) trace peptide (~3000 cpm) were then added and left to incubate for a further 22 h at 4°C. Free and bound CNPsp were then separated by solid-phase second antibody method (donkey anti-sheep SacCel, Immunodiagnostic Systems, Boldon, UK) in 2% polyethylene glycol/phosphate buffer (final Sac-Cel concentration 5%) at room temperature for 30 min. Tubes were then centrifuged at 2800 × *g* for 15 min, the supernatant decanted and pellet counted in a Gammamaster counter (LKB, Uppsala, Sweden). CNPsp immunoreactivity is neither altered by hemolysis up to 8 g/L nor by plasma lipid at up to 15 g/L. The limit of sample detection for this assay is 5 pmol/L, an ED50 of 284 pmol/L and a 99th percentile upper limit of normal range of 130 pmol/L (*n* = 109). The intra-assay CV is <7% with inter-assay CVs at 22% at 200 pmol/L and 11% at 780 pmol/L (3).

proBNP was determined by in-house immunoassay (7, 8). TnI was determined by a late generation assay (Abbott Architect) with a 99th percentile cut-off of 0.03 μg/L. Investigational TnT values were determined using a high-sensitivity assay (Elecsys 2010 analyzer, Roche Diagnostics) with a 99th percentile cut-off of 14 pg/mL. All hsTnT results were submitted to Penzberg during the worldwide reassessment of hsTnT by Roche (9), and only three required adjustment, all of which were below 14 pg/mL.

### Statistics

Continuous variables are presented as median [interquartile range (IQR)] whereas categorical variables are numbers and percentages. Continuous variables were analyzed by Mann–Whitney *U* test and categorical variables by Pearson χ^2^ test. Relational analysis of plasma analyte concentrations using Spearman rank order correlation testing and receiver operator curve (ROC) analysis were carried out using SPSS v22 (IBM). For ROC curve generation and biomarker panel comparisons, biomarker data were analyzed as descriptive standardized variables (*z*-scores). In all cases, the standardized variable was derived from the maximum biomarker value obtained from the *t* = 0, 1, and 2 h samples, i.e., the maximum of the three values. ROC curve comparisons were done according to Hanley and McNeill (10). In all analyses, a *P*-value <0.05 was considered significant.

## Results

### Patient characteristics

A total of 494 patients were enrolled in this study. Demographic data for this group are given in Table 1. Approximately 23% of patients had final adjudicated diagnosis of myocardial infarction (MI), 8% had definitive unstable angina (UA), 5% had other cardiac disorders (such as arrhythmia, sick sinus syndrome, heart failure), and 64% had chest pain of non-cardiac origin. All patients completed 45- and 365-day follow up. Respectively, mortality rates at 45 and 365 days were 1% (*n* = 4) and 4% (*n* = 18); subsequent MI 2% (*n* = 10) and 5.7% (*n* = 28); subsequent episode of ADHF 0.6% (*n* = 3) and 2% (*n* = 10); subsequent stroke 1% (*n* = 4) and 2% (*n* = 10). The composite end-point of death or MI at 365 days was 9% (*n* = 44).

**Table 1:** **Baseline data for the prospective chest pain group (median, IQR, or percent)**.

	Myocardial infarction (MI)	Unstable angina (UA)	Other cardiac disorder	Non-cardiac chest pain	All patients	*P*-value
**Patient, no**. (%)	112 (23)	39 (8)	24 (5)	319 (64)	494 (100)	
**Gender, no**. (%)						
Male	76 (68)	25 (64)	14 (58)	181 (57)	296 (60)	
Female	36 (32)	14 (36)	10 (42)	138 (43)	198 (40)	
**Age (years)**						
Male	66 (56–76)	64 (58–70)	64 (52–77)	59 (48–70)	62 (51–70)	
Female	77 (68–86)	66 (59–73)	72 (65–80)	69 (58–80)	69 (59–80)	
**Analytes**						
Chol (mg⋅dL^−1^)^a^	180 (154–216)	172 (141–213)	183 (157–201)	189 (154–215)	185 (154–216)	
HDL (mg⋅dL^−1^)^a^	41 (34–50)	38 (35–42)	41 (30–58)	44 (37–53)	42 (36–51)	
LDL (mg⋅dL^−1^)^a^	112 (93–143)	104 (77–139)	100 (84–124)	112 (89–135)	112 (89–135)	
Trig (mg⋅dL^−1^)^b^	142 (97–195)	128 (95–177)	142 (88–181)	124 (97–186)	133 (97–186)	
BMI (kg⋅m^2^)	27.7 (24.7–31.3)	27.2 (25.1–30.3)	27.7 (24.6–31.8)	27.7 (24.9–31.3)	27.7 (24.7–31.1)	
**Risk factor** (%)						
Hypertension	78 (70)	33 (85)	20 (83)	192 (60)	323 (65)	
Diabetes	19 (17)	9 (23)	4 (17)	43 (13)	75 (15)	
Current smoker	16 (14)	2 (5)	0 (0)	44 (14)	62 (13)	
Ever smoker	56 (50)	21 (54)	19 (79)	157 (49)	253 (50)	
**History** (%)						
CVD	78 (70)	36 (92)	13 (54)	199 (62)	326 (65)	
MI	36 (32)	20 (51)	10 (42)	99 (31)	165 (33)	
CABG	9 (8)	6 (15)	3 (13)	35 (11)	53 (10)	
Hyperlipidemia	62 (55)	34 (87)	14 (58)	193 (61)	303 (60)	
Angina	48 (43)	30 (77)	17 (71)	158 (50)	253 (50)	
Heart failure	10 (9)	4 (10)	2 (8)	33 (10)	49 (10)	
**ECG results** (%)						
LBBB	2 (2)	1 (3)	1 (4)	7 (2)	11 (2)	
ST-elevation	22 (20)	0 (0)	2 (8)	0 (0)	24 (5)	
ST-depression	10 (9)	1 (3)	3 (13)	2 (1)	16 (3)	
T-wave inversion	20 (18)	5 (13)	6 (25)	30 (9)	61 (12)	
No change	56 (50)	31 (79)	14 (58)	281 (88)	382 (78)	
**Presentation marker levels**						
hsTnT (ng/L)	79 (37–219)	6 (3–10)	22 (8–36)	5 (3–12)	8 (3–27)	<0.01
TnI (ug/L)	0.25 (0.07–1.30)	0.01 (0.01–0.01)	0.02 (0.01–0.04)	0.01 (0.01–0.01)	0.01 (0.01–0.03)	<0.01
CNPsp (ng/L)	51.8 (45.7–67.3)	51.6 (42.1–62.0)	54.3 (46.5–66.4)	50.1 (42.3–62.2)	50.6 (42.7–63.1)	NS
NTproBNP (ng/L)	87 (32–166)	50 (33–166)	133 (51–231)	39 (18–88)	45 (21–122)	<0.01

*^a^To convert mg⋅dL^−1^ cholesterol to mmol/L, multiply by 0.0259*.

*^b^To convert mg⋅dL^−1^ triglycerides to mmol/L, multiply by 0.0113*.

### Presentation levels of CNPsp, proBNP, and troponin

**Figure 1:**
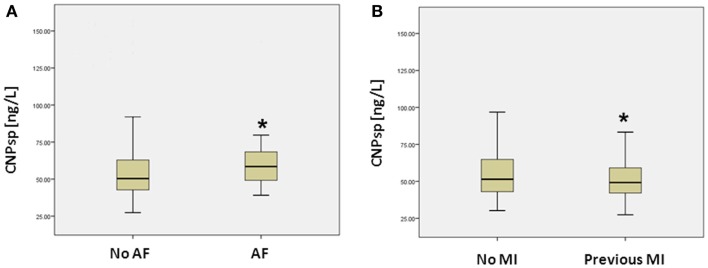
**Plasma concentrations (median, IQR) of CNPsp (ng/L) in patients with and without atrial fibrillation (AF) (A) and those with and without a previous history of MI (B)**. *Indicates statistical significance (*P* < 0.05) by non-parametric testing.

Presentation CNPsp levels had significant positive correlations with ECG ST-segment depression (*r* = 0.105, *P* < 0.05), HDL (*r* = 0.132, *P* < 0.01), and sodium (*r* = 0.116, *P* < 0.05), but had significantly negative correlations with respiratory rate (*R* = −0.104, *P* < 0.05) and plasma creatinine (−0.125, *P* < 0.01). CNPsp had no correlation with proBNP, TnI, or hsTnT. Plasma levels of hsTnT, TnI, proBNP, and CNPsp dissected by adjudicated diagnosis are shown in Table 1. Whereas proBNP, hsTnT, and TnI levels were clearly and significantly elevated in patients suffering either MI or other cardiac disorders (Table 1, *P* < 0.01), CNPsp levels were not changed in these groups. Instead, CNPsp levels were significantly elevated in those patients whom had evidence of atrial fibrillation (AF, *n* = 19) during their emergency department presentation [median AF, 58.5 (48.6–69.3) vs. median non-AF 50.4 (42.7–63.0), *P* < 0.05, Figure 1A] whereas they were significantly lower in those with a history of previous MI [median previous MI (*n* = 153), 47.8 (40.7–56.9) vs. non-MI (*n* = 341), 49.3 (41.9–64.3) *P* < 0.05, Figure 1B]. Of note, median CNPsp levels in AF patients without a history of MI (*n* = 11, 60.2 pmol/L) were higher than those in patient with a history of MI (*n* = 8, 57.0 pmol/L).

### ROC analyses of CNPsp vs. other cardiac markers

**Figure 2:**
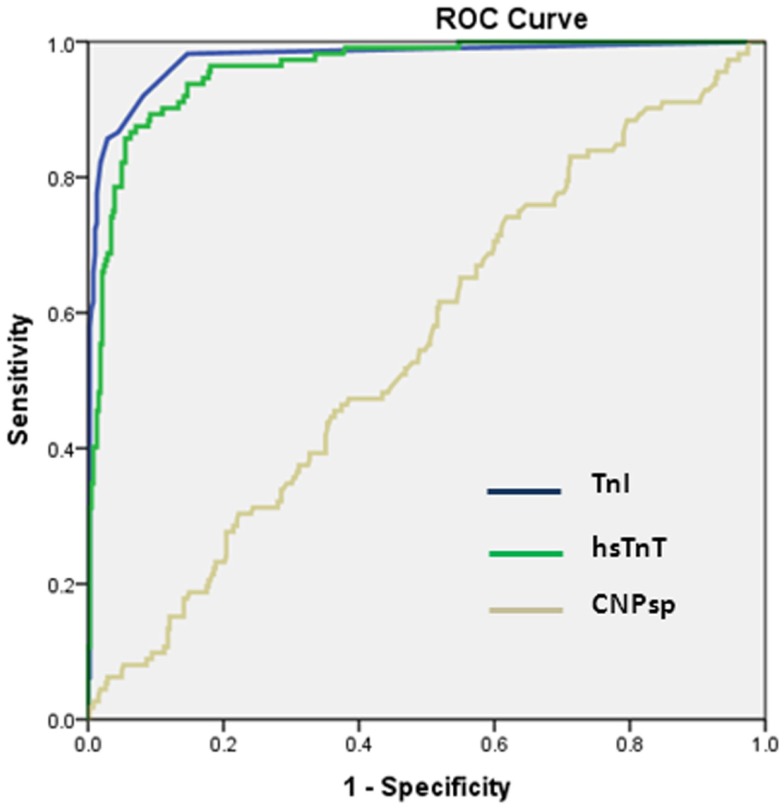
**Receiver operator curve curves for the identification of MI (*n* = 112) in the chest pain study group**. Clinical TnI and investigational hsTnT assays performed similarly (both *P* < 0.001). CNPsp measurement did not generate a significant AUC (0.56, *P* = 0.07) for the identification of MI and did not add to TnI or hsTnT measurement.

As expected, cardiac TnI (AUC = 0.974, 95% CI 0.957–0.992, *P* < 0.001) and hsTnT (AUC = 0.959, 95%CI 0.941–0.977, *P* < 0.001) had excellent ability to identify acute MI (Figure 2). By contrast, CNPsp did not generate significant ROC curves for any of MI (AUC = 0.56, 95% CI 0.496–0.614, *P* = 0.07, Figure 2), UA (AUC = 0.51, *P* = 0.518) or alternate cardiac disorders (AUC = 0.59, *P* = 0.16). Furthermore, CNPsp concentrations did not add to AUC or specificity/sensitivity values for either troponin.

### Prognostic ability of CNPsp

**Figure 3:**
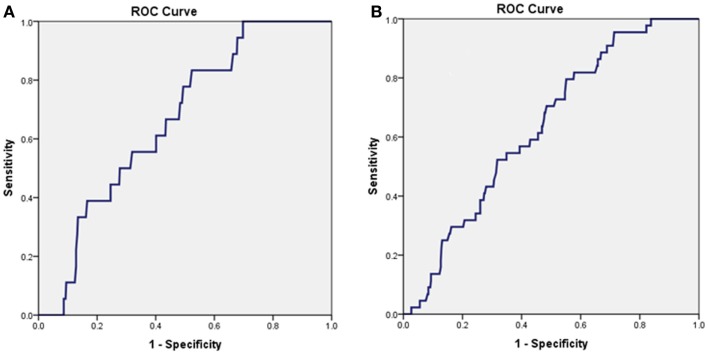
**Presentation CNPsp levels lower than 52 ng/L could generate significant AUC curves for the identification of death within 1 year (*n* = 18, AUC = 0.663, *P* = 0.019) (A) and the composite end-point of death/MI within 1 year (*n* = 44, AUC = 0.631, *P* < 0.01) (B)**.

In the whole study group, a presentation concentration of CNPsp <52 ng/L predicted death (*n* = 18) at 1 year (AUC = 0.663, 95% CI 0.563–0.764, *P* = 0.019, Figure 3A) and also predicted a composite end-point of death/MI (*n* = 44) at 1 year (AUC = 0.631, 95% CI 0.560–0.706, *P* < 0.01, Figure 3B). Combining CNPsp with proBNP improved the proBNP AUC for 1 year mortality from 0.77 to 0.82 and the composite end-point of death/MI AUC from 0.73 to 0.77, but neither of these improvements were significant.

In patients suffering MI (*n* = 112), CNPsp alone did not generate a significant AUC for any future event. However, adding CNPsp to proBNP improved the proBNP AUC for MI within one year (*n* = 12) from 0.647 (95% CI, 0.488–0.805, *P* = 0.098) to 0.707 (95% CI, 0.563–0.850, *P* = 0.020). In non-MI patients (*n* = 382), addition of CNPsp improved the AUC of proBNP for prediction of death at 1 year (*n* = 12) from 0.766 to 0.833 (95% CI, 0.747–0.920, *P* < 0.001), again a non-significant improvement.

## Discussion

The major findings of this work are (i) CNPsp does not identify ACS in patients with chest pain or those with alternate disorders such as myopathies/valve disease as assessed by ROC AUC analysis; (ii) CNPsp levels are significantly elevated in patients presenting with AF, yet significantly lower in those with previous history of MI; (iii) if CNPsp measurement has any utility at all in ACS, it may possibly be found in assisting NT-proBNP-based risk assessment of future major adverse events.

The lack of CNPsp to generate ROC significance in MI is consistent with our earlier data, which indicated that CNPsp did not display much of a dynamic range in patients suffering STEMI (3). Comparison with CNP and NT-proCNP is difficult as there are few reports that have described the acute response of CNP or NT-proCNP during MI. CNP has been studied loosely in the context of MI patients (11), did not show much dynamism in the early hours after MI and also displayed less powerful prognostic ability up to 2 years post-MI, compared with BNP and ANP. Part of this point of difference probably largely stems from the ubiquitous nature of CNP secretion, which comes from multiple sources, not just cardiac (4) and also that circulating CNP concentrations tend to be much lower compared with ANP and BNP. In this regard, CNPsp tends to display a secretion pattern more like that of NT-proCNP in that renal and cardiac venous drainage contains highest amounts of both peptides (3, 4), but this still does not translate into useful performance characteristics in chest pain patients. Combining this secretion profile with the weak, but significant, negative correlation with plasma creatinine, it seems probable that CNPsp concentrations are markedly influenced by renal status.

The underlying mechanism and relevance of elevations in CNPsp concentrations in patients with AF is unclear but is consistent with our previous finding of a significant correlation of arterial CNPsp with heart rate (3). Both BNP and CNP can increase heart rate *in vitro* as well as increasing L-type Ca^2+^ channel activity and the hyperpolarization-activated current *I*(*f*), an effect which is reliant upon phosphodiesterase-3 presence and activity (12). Thus, whether elevated circulating CNPsp concentrations in the setting of AF represents a passive response or is indicative of a more defined biological relationship with heart rate is a potential area for further study. Further, we cannot exclude the potential for confounding multiple comparison issues as affecting this result.

The ability of CNPsp to identify those at risk of death within 1 year and potentially improve the AUC of NT-proBNP identification of mortality risk is an interesting exploratory finding. Although the data are only from a very small group, and findings can only be considered as preliminary, they do suggest that CNPsp, like CNP, is worthy of further study in risk prediction in patients suffering chest pain. In particular, the CNPsp AUC for mortality (0.66) is close to the 2-year mortality predictive AUC of highly sensitive TnI assays (~0.70), which were not used here (13). Further studies will need to address any potential for additive utility between CNPsp and hsTnI. A single study has suggested CNP does not appear to predict well either death or re-infarction in MI sufferers (11) but like our present report, suffered from small numbers. CNP does, however, have strong associations with left ventricular fibrosis and subsequent systolic and diastolic impairment (14), the development of arterial stiffness, endothelial function, and atherosclerosis (15) and the preservation of long-term vascular and renal function (16). Thus, future studies assessing the comparative risk prediction abilities of CNP, NT-proCNP, and CNPsp in suitably sized chest pain populations will need to take into account vascular and renal contributions to disease progression.

In conclusion, CNPsp measurement is unlikely to have utility in assisting the diagnosis of MI (either STEMI or NSTEMI) or UA in patients presenting with chest pain. CNPsp concentrations are elevated in patients presenting with AF and the potential biological relevance of this should be further determined in appropriate experimental models. Finally, CNPsp concentrations are lower in chest pain patients with a history of previous MI and this feature may have potential to assist in the prognosis of subsequent mortality or MI. Appropriately designed study groups that take into account vascular and renal contributions as well as perform head to head comparisons with CNP and NT-proCNP, will be needed to properly address this.

### Limitations

First, our study size is satisfactory for assessing potential diagnostic ability of CNPsp with respect to MI and UAP, but it can only provide exploratory data for prognostic implications and thus any findings are interpreted accordingly. Second, our adjudication of MI relied on a conventional TnI assay. Incorporation of a high-sensitivity troponin I assay may have provided subtle differences in analyses (17). Third, whereas the half-life and clearance of pro-CNP forms are known (18), such data for CNPsp and other signal peptides are not available.

## Author Contributions

CP, MR, RT, and MT, designed research; CP, JL, MT, and SA, performed research; CJP, MR, and RT, analyzed data; and CP, RT, MR, MT, SA, and JL, wrote the paper.

## Conflict of Interest Statement

The University of Otago, New Zealand has filed a patent application on the composition and diagnostic/prognostic use of CNPsp measurement in cardiovascular and cardiorespiratory disorders. Chris J. Pemberton and Mark Richards are listed as inventors on this application. All other authors have nothing to disclose.

## Abbreviations

AF, atrial fibrillation; CNP, C-type natriuretic peptide; CNPsp, C-type natriuretic peptide signal peptide; STEMI, ST-elevation myocardial infarction; UA, unstable angina.
